# Letter from Professor J. Allen

**Published:** 1852-01

**Authors:** J. Allen


### ARTICLE IX.

### *Letter from* PROFESSOR J. ALLEN, *to the senior Editor, dated, Cincinnati, Dec.* 15*th*, 1851.

DEAR SIR: I have the pleasure of informing you, that I have so far perfected my improved method of mounting artificial teeth upon metallic plates, that I have adopted it exclusively in my practice, which I find much more satisfactory to myself and my patients, than any other method I have heretofore known. This improvement consists in uniting single teeth to each other, and to metallic plates, in such a manner as to form a most perfect and continuous gum upon artificial dentures, without the slightest check or crack. It has been subjected to the severest tests during the past two years, but more especially, within the last year, by myself and others, as there are now some twenty dentists, in different parts of the union, who have adopted this method in their practice, all of whom have expressed their decided preference for this style of work. I intend soon to send you some specimens of this improvement, for the Baltimore College of Dental Surgery, if they would be acceptable.

